# Dance Is for All: A Social Marketing Intervention with Children and Adolescents to Reduce Prejudice towards Boys Who Dance

**DOI:** 10.3390/ijerph18136861

**Published:** 2021-06-26

**Authors:** Ana Silva, Elisabete Sá, Joaquim Silva, José Carlos Pinho

**Affiliations:** 1 School of Economics and Management, University of Minho, 4710-057 Braga, Portugal; alsilva@ipca.pt (A.S.); jcpinho@eeg.uminho.pt (J.C.P.); 2 Instituto Politécnico do Cávado e do Ave, 4750-810 Barcelos, Portugal; 3 Interdisciplinary Centre of Social Sciences (CICS.NOVA.UMinho), School of Economics and Management, University of Minho, 4710-057 Braga, Portugal; josilva@eeg.uminho.pt

**Keywords:** prejudice in dance, social marketing intervention, children and adolescents, male dancers, boys who dance, gender

## Abstract

Dance is proven to offer a myriad of physical, psychological, and social benefits. However, because dance has been frequently perceived as a feminine practice, there is a prevailing prejudice towards boys who dance, making it hard for them to engage in this physical activity. Social marketing has been presented as a promising framework to deal with different social problems, including prejudice, although its effectiveness is still difficult to establish. Drawing on the Theory of Planned Behavior (TPB), a quasi-experimental study involving a sample of 436 children and adolescents, composed of 51.38% boys and 48.62% girls was implemented to measure the effectiveness of a Social Marketing Intervention (SMI) in reducing prejudice towards dance and boys who dance, in particular, and in increasing their intentions to practice this physical activity. The study furthermore aimed to compare the influence of the SMI on participants of two different stages of child development to ascertain when it is most effective to intervene. The questionnaire was used to collect information and included items derived from relevant literature. To assess differences between children and adolescents before and after the SMI, the analysis relied on independent *t*-tests and paired *t*-tests. Results suggest positive effects of the SMI on some dimensions of the TPB.

## 1. Introduction

Normative gender associations often place dance into the feminine domain and establish an association with homosexuality [1,2,3,4]. Consequently, boys who dance are viewed as effeminate, homosexual, and not real men [5]. Some authors acknowledge that there are greater manifestations of sexual prejudice when masculinity is threatened [6]. The existence of negative attitudes, encapsulated in prejudice, will likely influence individuals not to perform specific behaviors [7,8,9]. This is the case of prejudice towards dance and, particularly, towards boys who dance, which has been identified as an inhibitor for children and adolescents to join dance activities [10]. Due to the negative attitudes embedded in such prejudice, young people may miss the opportunity to seize the different health benefits of a physical fitness program achieved through dance [11,12,13,14]. The development of positive attitudes will likely impact the willingness to participate in some physical activities, as is the case of dance [15,16]. 

Research suggests that prejudice is age-related [17,18]. Particularly, the prejudice towards boys who dance tends to be more expressive between the ages of 11 and 16 years old [19]. It has been considered that changing attitudes is easier in earlier stages of development, and for this reason, childhood is regarded as an important period to prevent and fight prejudice [20,21]. Adolescence is also an important stage to develop positive attitudes [22], and it is a period of life when it is possible to change attitudes, when social relationships are used as influencers [17]. Therefore, children and adolescents are important targets for interventions aiming at fighting prejudice by changing attitudes and behaviors [23], particularly through Social Marketing Interventions [24]. There are indications that age and development effects reduce from childhood to adolescence, inasmuch that they are replaced by social influences [18,22,25]. Therefore, age-related differences should be integrated into the analysis [17,18,23].

Social Marketing Interventions (SMI) have been showing effectiveness in promoting positive behaviors, including the practice of physical activity, and in reducing unhealthy behaviors, and have been targeting different age groups, namely, children, young people and adults [26,27]. However, there is a scarcity of research focusing specifically on the role of SMIs in fighting prejudice [24], which may prevent the involvement in healthy physical activities. Thus, the study aims to explore the effects of an SMI designed to reduce young people’s prejudice towards dance, and particularly towards boys who dance and to increase their intentions to engage in dance activities. Moreover, it aims to compare those effects among two different stages of child development, namely, children and adolescents.

The research draws on the Theory of Planned Behavior (TPB), which has been considered as an appropriate theoretical framework for designing behavior change interventions in different contexts, such as the case of physical activity [28]. Ajzen [7] points out, as the main assumption of the TPB, the fact that people will perform a behavior when they (i) evaluate it positively (attitude), (ii) believe that significant others want them to engage in it (subjective norm), and (iii) consider that this behavior is under their control (perceived behavior control). Interventions based on the TPB framework aim to change behavioral, normative, and/or control beliefs, consequently motivating the performance of the behavior [28].

Attitudes are beliefs about a particular behavior and reflect the expectations and assessments about that behavior [29]. Prejudice is a negative attitude that is most noticeable between the ages of 12 and 16 years [30]. However, the literature suggests that it is easier to change attitudes in earlier stages of development [20,21]. Thus, it is expected that an SMI has a positive influence on the attitudes towards dance and towards boys who dance [31,32], and that this influence may be greater on children than on adolescents [20,21]. Accordingly, the following hypotheses are put forth:

**Hypothesis** **1** **(H1).** *The average score of attitudes towards dance will differ before and after the SMI, and this difference will be greater in children than in adolescents*.

**Hypothesis** **2** **(H2).** *The average score of attitudes towards boys who dance will differ before and after the SMI, and this difference will be greater in children than in adolescents*.

Subjective norms regard how individuals’ behaviors are viewed by others [7]. This construct is related to the support to perform a behavior received from significant people, such as family, friends or other influencers [8]. As there is evidence that peer pressure is greater in adolescence [19,33,34], the authors predict that an SMI influence in perceived subjective norms [35] will be different among the two child development stages. Thus, we posit that:

**Hypothesis** **3** **(H3).** *The average score of the subjective norms about boys who dance will differ before and after the SMI, and this difference will be greater in children than in adolescents*.

The perceived behavior control comprises the degree to which a person feels capable and confident of executing the desired behavior and the extension to which a person considers that the performance of the behavior is easy or difficult [7,29]. Beliefs related to perceived behavior control regard factors that may facilitate or hinder the performance of the behavior [36], including the perceived ability to perform dance activities. SMIs that allow participants to experiment their abilities may contribute to improving these beliefs [37,38]. However, this influence may differ according to the child development stage, considering, for example, that the levels of motor coordination tend to improve towards adolescence [39]. Hence, the following hypothesis is suggested:

**Hypothesis** **4** **(H4).** *The average score of the perceived behavior control related to dance will differ before and after the SMI, and this difference will be greater in adolescents than in children*.

According to the TPB, the intention is an indicator of how much people aim to perform the desired behavior [29], being a strong predictor of a physical activity behavior [40]. In addition to the mentioned influencers of intention, when focusing on children and adolescents, it is important to consider the degree of autonomy to make decisions about a behavior. Parents have more influence on the decision-making process at an earlier age [1,41,42], whereas in adolescence, individuals start to win more independence in the decision-making process [43]. Therefore, the authors predict that:

**Hypothesis** **5** **(H5).** *The average score of the intention to join dance activities will differ before and after the SMI, and this difference will be greater in adolescents than in children*.

## 2. Method

### 2.1. Study Design

An experimental research design was planned to test the formulated hypotheses with pre-test and post-test data collection, including experimental and control groups. This design is appropriate to the study’s purpose, as it allows to verify the cause–effect relationship of an intervention that is evaluated by its influence on the variables of the model among an experimental and a control group of children and adolescents [44,45]. The study falls into a quasi-experimental design category [46,47] since it was performed with individuals organized in school classes, where the randomization of the subjects was not possible. The research was divided into three stages, (i) the implementation of a questionnaire; (ii) the implementation of the SMI to the experimental group; (iii) the re-implementation of the questionnaire. The goal was to measure the differences in the effect of the SMI on the TPB constructs, comparing children and adolescents. 

### 2.2. Social Marketing Intervention

As the intervention took place during the COVID-19 pandemic situation, it was delivered in the online format, pairing with the regular school activities that also migrated to this format during the lockdown. Although the situation imposed this format, the online environment to reach a younger target audience through social marketing campaigns is starting to be used in contexts such as fighting cyberbullying [48] and promoting physical activity [40]. In fact, campaigns that use the online channel may allow and facilitate the adaptation to young people’s language and behavior, contributing to the effectiveness of these campaigns [48]. The use of online campaigns in school initiatives regarding the prevention or combat of problems is also referred to as adding value [48].

The SMI was designed to fight prejudice towards dance and boys who dance, grounded on social marketing benchmarks [49,50] and based on relevant behavior change theories and methods [28,51,52]. The intervention used techniques designed to attain different key objectives, with the general aim of increasing awareness about dance as a physical activity suitable for everyone and conveying the idea that it is necessary to stop prejudice towards boys who dance. 

The SMI included four main modules that were pre-tested with children and adolescents to assess their suitability and understanding by the study’s target audience. In the first one, participants observed six posters showing boys dancing alone, in couples or group and delivering messages such as “Stop Prejudice”, “Stop Prejudice, dance is also for men”, and “Stop Prejudice, boys dance too”. The key objective of this module was that children and adolescents would start to be aware that dance is not only for girls, that it is for everyone and that it is necessary to stop prejudice. 

The second module consisted of a three minutes and 27 s video titled “Dance is for all”, in which participants got access to testimonials from men and boys currently dancers or former dancers (one ballet dancer, one former dancesport dancer, one former dancer, football player and football coach, one dancesport dancer, and one dance teacher). Through the video, children and adolescents got contact with role models and received first-person feedback about what dance meant to them, the benefits of dance and also the problems that they had to face due to prejudice regarding boys who dance, to promote learning through vicarious experience [53].

The third module consisted of a second video, with two minutes and 43 s duration, titled “Dance, a physical activity”, through which children and adolescents got access to two informative videos with different messages, such as “Dance is a physical activity”; “Dance can be practiced in solo, couple or group”; “Dance can also be a competitive sport”; “Dance is for boys, Dance is for girls, Dance is for all”; “Respect who dances”. The participants also were exposed to examples of the different types of dance. The key objectives were that they would be aware that dance is a physical activity, that it can have different styles and that it can be practiced by everyone, regardless of gender, to reinforce the posters’ messages.

The fourth module consisted of a third video with dance lessons comprising HipHop, Ballet and Dancesport (samba and chachacha). The latter style was included as it is considered a sport modality and there is evidence that when dance is considered as a sport it is more desirable for boys [10]. The key objective of this module was to promote learning from activity-based experience and increase self-efficacy [52,54], through the experimentation of dance and, with it, to raise awareness to dance as physical activity. At the end of each module, participants were asked to fill a control form to assess the awareness of the condition they were exposed to, to guarantee that those who reported having participated indeed did it and to collect preliminary feedback on the SMI. Regarding the dance experimentation, the majority of participants (75.00%), both children (75.58%) and adolescents (74.62%) considered hip hop their preferred style. Although ballet was chosen as the second preferred style by the participants (10.55%), when we analyzed only the boys’ preferences, ballet was on the bottom of the list. This is consistent with research that indicates that some dance styles are more associated with a normative view of masculinity, and for this reason, they are more appealing to boys [1]. After being exposed to some components of the intervention, however, 93.12% of the participants agreed that ballet can be practiced by both boys and girls, an even greater percentage than those who consider the same for hip hop (92.20%). 

As the authors wanted to assess whether the SMI would have different effects on children and adolescents, the same materials and activities were presented to both targets. This allowed controlling, as much as possible, the experimental conditions.

### 2.3. Sample

A sample consisting of 213 children and 223 adolescents, 51.38% boys and 48.62% girls was selected from two schools in the city of Braga, Portugal, following a convenience sampling procedure [55]. To better describe the target, the school cycle of the Portuguese educational system of the respondents was used as a proxy to distinguish children from adolescents. Specifically, children predominantly attend the school’s 2nd cycle and are aged between 10 and 12 years old; adolescents are enrolled in the school’s 3rd cycle and are aged between 13 and 16 years old [56,57]. This classification is based on the PedsQLTM (Pediatric Quality of Life Inventory), which distinguishes children, aged from 8 to 12 years old, from adolescents, aged from 13 to 18 years old [56,58,59]. Thus, each school’s cycle incorporates more homogeneous individuals in terms of development, and for this reason, the authors did not use the direct age of the participants to compare children and adolescents. The demographic characteristics of the participants are presented in Table 1.

### 2.4. Data Collection

Data were collected during the 2019/2020 school year. A questionnaire was developed using the online software Qualtrics and implemented in two phases (before and after the SMI). The questionnaires were delivered to the children and adolescents through the digital platforms used by the schools for the online classes. Only those children and adolescents who participated in both the first (pre-test) and second (post-test) moments were included in the sample. Although participation was not mandatory, parental consent was obtained for children and adolescents to participate in the research.

To minimize common method bias and maximize content validity, the authors used multiple strategies, such as multidimensional scales for each construct; simple language suitable for the target audience; pre-test of the questionnaire and feedback from children, academics and teachers of the target grades’ participants; positive and negative items were used in a balanced way; teachers were involved as intermediaries of the whole process [60,61,62,63,64]. Harman’s single factor test revealed that systematic variance was not a problem in the present study [65].

### 2.5. Measures

To test the hypotheses, the authors designed a questionnaire based on well-established scales that were adapted to suit the specific object of the study [19,66,67]. The attitude towards dance (Attd) was measured using seven items, adapted from the Schwarzer and Luszczynska’s [67] research, and was assessed with a non-comparative multi-item semantic differential scale (e.g., “Dance is an activity (Harmful—Beneficial)”; “Dance is an activity (Boring—Exciting)”). The other constructs were considered using a 5-point Likert scale, ranging from 1 = “Strongly Disagree” to 5 = “Strongly Agree”. The attitude towards boys who dance (Attb) was measured using 14 items adapted from Sanderson [19] (e.g., “When I see a boy dancing, I consider it a stupid situation”; “Boys should not practice dance”; “I don’t like to see guys expressing dance moves”). The subjective norms (SN) included 11 items derived and adapted from David and Rundle-Thiele’s [66] scale (e.g., “People who are important to me consider that boys should not practice dance”; “People who are important to me consider that boys who dance are effeminate”; “People who are important to me approve if I decide to choose dance as an activity for me to practice”). The perceived behavior control (PBC) comprised three items derived and adapted from the same scale (e.g., “If I want, I can choose dance practice”; “If I want, I can start to dance next month”). The last construct, the intention to join dance activities (INT), was measured from three items also derived and adapted from David and Rundle-Thiele’s [66] scale (e.g., “I intend to start practicing dance next school year”; “I will seek to participate in dance activities next school year”).

Since the data analysis required paring the sample to assess individual differences before and after the SMI, the children and adolescents were asked about their school year, number or letter of the class and individual student number. This information was kept confidential and was only used for the mentioned purpose.

### 2.6. Analysis

Data were analyzed using IMB SPSS 26.0 software. To examine the internal consistency of the adapted measures, the authors estimated the Cronbach coefficient alpha (α) for the entire scale and each of the specific measures. The authors further tested the internal consistency of the scales for the entire pre-test sample (before SMI) and each of the groups (control and experimental), repeating the procedure for the post-test sample (after SMI), to verify the consistency of the measures in reflecting the same underlying construct. The results (Table 2) indicate a good internal consistency of the measures, with the estimates exceeding the recommended threshold of 0.70 [68], which is in line with previous studies [66,67]. Only the construct subjective norms (SN) presented alphas slightly below the cut-off limit of 0.70 (from 0.568 to 0.719), as in the study by David and Rundle-Thiele [66] in which the alpha of SN was 0.66. The obtained alphas for SN are close to or above 0.60, which deemed an acceptable value for exploratory research [68]. Therefore, the authors decided to proceed with the analysis, even because the alphas of the entire scale were significantly above the thresholds, supporting the assertion of the internal consistency of the measures in different samples and moments.

As the primary goal of the study is to evaluate changes in children’s and adolescents’ perceptions before and after the SMI, and to compare the effects between these two groups, the analysis relied on paired *t*-tests and independent *t*-tests using the composite variables calculated as the means of the observable variables of each construct, similarly to previous research [69,70,71,72,73,74].

Normality tests, namely the Kolmogorov–Smirnov Test and the Shapiro–Wilk Test were computed. The results indicate that the *p*-value is lower than 0.05 for both tests, which means that the data does not follow a normal distribution [75,76]. However, as in social sciences a certain deviation from normality is acceptable [77], despite the results of the Kolmogorov–Smirnov Test and the Shapiro–Wilk Test, skewness’ values between ± 2/3, and kurtosis between ±7/10 are accepted [78]. Therefore, the authors performed the Skewness and Kurtosis tests, and all data are within the normality, since the higher values for the skewness of the experimental and control groups were −1.218 and −1.403, respectively, and for kurtosis of the experimental and control groups were 5.401 and 2.039, respectively. For these reasons, these values are considered acceptable to prove normal univariate distribution [78]. The results indicate that parametric tests can be used [79]. The literature stresses the advantages of using parametric tests (e.g., ability to control the Type I error rate and greater statistical power) instead of using the non-parametric tests [80]. Thus, the parametric tests were performed in this research. 

## 3. Results

The differences in perceptions were compared before and after the SMI and between children and adolescents and were evaluated in the experimental and control groups. Using the paired samples test, the authors compared the constructs before and after the SMI in the groups of children and adolescents separately. Concerning the children in the experimental group (Table 3), their attitudes towards dance, attitudes towards boys who dance, and subjective norms about boys who dance were significantly different (*p* ≤ 0.05) after the SMI, compared to the perceptions before this intervention. For these constructs, on average, children were more positive (M_AFTER_ > M_BEFORE_) towards the TPB dimensions after the SMI, although some changes were also verified in the control group.

Regarding the adolescents of the experimental group (Table 4), the constructs attitudes towards dance and intention to join dance activities were significantly different (*p* ≤ 0.05) after the SMI, compared to the perceptions before this intervention, which were not verified in the control group. Therefore, in those constructs, adolescents were more positive (M_AFTER_ > M_BEFORE_) after the SMI than they were before.

The results indicate that the SMI had some effect on children’s and adolescents’ perceptions and suggest that it affected differently the two groups. To examine whether there are significant differences between children and adolescents in their perceptions’ change, the authors performed an Independent Samples Test. The results (Table 5) show that, in the experimental group, while before the SMI the differences between children and adolescents were not statistically significant (*p* > 0.05) for all the constructs, after the intervention, statistically significant differences were observed (*p* ≤ 0.05) in the attitudes towards dance and in the subjective norms about boys who dance. It is relevant to highlight that in the control group, after the intervention, those variables did not show any significant differences (Table 6), strengthening the results of the experimental group. 

Thus, after the intervention, children tend to have more positive (M_Children_ > M_Adolescents_) attitudes towards dance and more positive (M_Children_ > M_Adolescents_) perceptions regarding subjective norms about boys who dance than adolescents. Although the effect sizes [81] are small in the experimental group, they increase after the SMI, compared with results before the SMI. Concerning the control group, an increase in the effect size is also verified, except for the subjective norms and the perceived behavior control, where the opposite occurs.

Concerning the control group, the construct attitudes towards boys who dance also has significant differences between children and adolescents before and after the SMI, as there are differences in the intention to join dance activities between children and adolescents (Table 6). This result is unexpected, insofar that the intervention was implemented only on the experimental group. However, the online context favors elements that the authors cannot control.

## 4. Discussion

As hypothesized, the average scores in all variables were, indeed, different after the SMI, with higher values reflecting a positive effect of the intervention. This result was verified for both children and adolescents. In the case of children, the SMI had a statistically significant positive effect on more variables, namely, the attitudes towards dance, attitudes towards boys who dance and the subjective norms. In the case of adolescents, although with lower expression, the intervention also affected the attitudes towards dance. As expected, adolescents’ intention to join dance activities was significantly improved, but not that of children’s, possibly because adolescents are more independent in their decisions than children [1,41,42,43]. Although it was only statistically significant for three variables in the children’s group and two variables in the adolescents’ group, this result offers a relevant contribution to support the argument of the effectiveness of the SMI. As only one of these variables was common to both groups (attitudes towards dance), the results suggest different effects of the SMI on these two levels of development. 

In fact, before the SMI, no significant differences were found between children and adolescents of the experimental group for any of the TPB constructs, but after the intervention, statistically significant differences in two out of the five constructs were identified. First, children come to perceive dance, as physical activity, even more positively than adolescents, which is consistent with the idea that it is easier to fight prejudice at earlier ages [20,21]. This result also reinforces the argument that negative attitudes towards dance are more expressive in adolescence [30]. Thus, the authors accepted H1, which suggested that attitudes toward dance would be different after the intervention and this difference, reflecting more positive attitudes, would be greater in children than in adolescents. 

Second, the SMI had a significant impact on children’s subjective norms, although not on the adolescents’, who significantly differ from children regarding this variable. Although both children’s and adolescents’ subjective norms had higher scores after the SMI, this change was significantly greater in children, as predicted, leading to accepting H3. Peer pressure may have had an important role here, as it is stronger in adolescence [19,33,34]. 

Although there was an increase in the perceptions that followed the hypothesized pattern regarding the attitudes towards boys who dance and the intention to join dance activities, the perceived behavior control had a larger increase in children, contrarily to what was predicted, and the differences between children and adolescents were not statistically significant for any of the three variables, leading the authors to reject hypotheses H2, H4 and H5. These results are interesting because they show that, despite the different effects that the SMI had on these two age development groups when analyzed in isolation, and despite the inherent differences these two targets might have, regarding some of the variables they did not differ substantially. Taken together, the results of this study suggest that children and adolescents seem to have some commonalities that would lead them to respond similarly to some of the stimuli of the SMI. However, the identified differences provide indications that an SMI with the purpose of the one used in the research may need to be adapted to the two different targets, so as to increase its effectiveness. This adaptation may be justified since not only the prejudice tends to have different expression in different ages and there is evidence that it is easier to change attitudes in earlier stages of development [20,21,30], but also the information processing and stimuli response change throughout childhood and adolescence [82], posing challenges to a standardized SMI for all children.

## 5. Conclusions

This study contributes to a deeper understanding of the effect of an SMI on children’s and adolescents’ perceptions of dance as physical activity and the differences between these two age development groups, assessed in the light of the TPB framework. The results of this study allow drawing two relevant conclusions. First, all the five variables analyzed scored higher after the SMI. Although only three were statistically different in the case of children, and two in the case of adolescents, both groups have in common the fact that they were significantly affected in their perception of dance as physical activity. Moreover, children’s perceptions of boys who dance were significantly more positive after the SMI. This is a relevant implication of the study, adding evidence of the power of social marketing to tackle social problems, particularly prejudice, which has been advocated by several authors [24,27,83,84]. The use of social marketing tools to fight prejudice towards dance as physical activity and towards boys who dance is an opportunity to accomplish this purpose, while also allowing to analyze the role of the SMIs outside the health field, where it has been most often applied [26,27,83,85]. Second, when the differences between the SMI effects on the children’s and adolescents’ groups are explored, it was concluded that they had similarities but also significant differences. These mixed results suggest that an SMI that is more adapted to the different targets may be beneficial. This study contributes to the work of educators and policymakers by suggesting that prejudice regarding some physical activities should be tackled differently at different ages and that earlier ages are more susceptible to change attitudes. Yet, future research should include younger children than those who participated in this study, to further explore this conclusion.

The mixed results found, both regarding the effect in each age development group in isolation and when comparing them, may be explored in future research. For example, this study did not make an in-depth approach to the parents’ influence. However, the literature suggests that parents, specifically, fathers, are an important influence on the normative beliefs and, particularly, on the type of prejudice addressed by this research [4,86,87,88]. It was found that the subjective norms of children were affected by the SMI, but not those of adolescents. Thus, it would be relevant to explore whether fathers have a greater influence on adolescents’ subjective norms about boys who dance. Parents were not targeted in this study, but future studies may also include them as a target for a specific SMI, to explore this influence on the adolescents’ perceived support.

Another possible influence that would benefit from further exploration is the general social view of dance. The use of the term “sport” instead of “art” to categorize the dance activity has been presented as an attempt to attract more male practitioners [89]. In this context, schools may also have an important role. The fact that most schools do not promote dance activities as a school sport or in physical education classes has a strong influence on children’s and adolescents’ preferences and choices [1,90]. Additionally, the way dance, and particularly boys who dance, are seen within different cultural contexts may also be important to consider, as culture plays an important role in the development and persistence of prejudice [91]. Attitudes towards boys who dance are rooted in social and cultural beliefs about gender roles regarding some activities [10,92,93]. This study was developed in a Western culture country, where sports are predominantly associated with football, which was historically stereotyped as a male activity [94,95], and dance is viewed as a feminine cultural practice [96]. Thus, a cross-cultural study could be relevant to assess such influences.

Besides the variables drawn from the TPB model, others could also be relevant to take into account in future research. For example, concepts such as perceived self-efficacy, which is influenced by socio-structural factors—consisting of economic conditions, socioeconomic status, educational structure, and family status—may indirectly influence behavioral effects [97]. These factors were not included in this study, but they might be relevant to understand the SMI effectiveness. Finally, some variables could not be controlled using the research design that was implemented, particularly when targeting young people and involving activity-based experience within the online environment used for the implementation. This limited control may help explain some differences found in the control group. Thus, implementing the designed intervention in a face-to-face context may also be necessary to compare the levels of SMI effectiveness, considering the implementation medium.

## Figures and Tables

**Table 1:** Demographic characteristics of the participants.

Characteristics	Total		Control Group		Experimental Group	
	Freq	%	Freq	%	Freq	%
Gender						
Male	224	51.38	104	50.98	120	51.72
Female	212	48.62	100	49.02	112	48.28
Level of child development						
Children (10–12 years old)	213	48.85	98	48.04	115	49.60
Adolescents (13–16 years old)	223	51.15	106	51.96	117	50.43
Nationality						
Portuguese	402	92.20	184	90.20	218	94.00
Other countries	34	7.80	20	9.80	14	6.00

**Table 2:** Internal consistency of the measures (Cronbach alpha).

Constructs	Total		Control Group		Experimental Group	
	Before SMI	After SMI	Before SMI	After SMI	Before SMI	After SMI
	α	α	α	α	α	α
ATTd (7 items)	0.915	0.926	0.907	0.921	0.921	0.930
ATTb (14 items)	0.846	0.838	0.822	0.848	0.861	0.830
SN (11 items)	0.669	0.658	0.568	0.617	0.719	0.684
PBC (3 items)	0.763	0.759	0.771	0.748	0.756	0.759
INT (3 items)	0.916	0.921	0.908	0.934	0.923	0.912
Entire scale (38 items)	0.906	0.884	0.893	0.879	0.913	0.888

**Table 3:** Paired samples’ test results of children and adolescents: differences before and after the SMI—children.

**Children Experimental Group**									
**Constructs**	**Before**			**After**					
	**M**	**SD**	**N**	**M**	**SD**	**N**	**t**	**df**	**Sig.** **(2-Tailed)**
ATTd	3.69	0.995	115	4.05	0.892	115	−4.680	114	≤0.001
ATTb	3.97	0.691	115	4.30	0.581	115	−5.253	114	≤0.001
SN	2.90	0.689	115	3.13	0.640	115	−3.355	114	≤0.001
PBC	3.22	1.302	75	3.51	1.201	75	−1.626	74	0.108
INT	1.90	1.168	75	2.12	1.201	75	−1.67	74	0.099
**Children Control Group**									
**Constructs**	**Before**			**After**					
	**M**	**SD**	**N**	**M**	**SD**	**N**	**t**	**df**	**Sig.** **(2-Tailed)**
ATTd	3.74	0.929	98	3.93	0.903	98	98	97	0.010
ATTb	3.97	0.625	98	4.27	0.537	98	98	97	≤0.001
SN	3.01	0.568	98	3.20	0.552	98	98	97	0.003
PBC	3.59	1.240	65	3.87	1.089	65	65	64	0.031
INT	2.03	1.161	65	2.08	1.069	65	65	64	0.722

Labels: M = mean; SD = standard deviation; ATTd = attitudes towards dance; ATTb = attitudes towards boys who dance; SN = subjective norms about boys who dance; PBC = perceived behavior control; INT = intention to join dance activities. Perceptions ranged from 1 (strongly disagree) to 5 (strongly agree).

**Table 4:** Paired samples’ test results of children and adolescents: differences before and after the SMI—adolescents.

**Adolescents Experimental Group**									
**Constructs**	**Before**			**After**					
	**M**	**SD**	**N**	**M**	**SD**	**N**	**t**	**df**	**Sig.** **(2-Tailed)**
ATTd	3.58	1.064	117	3.77	1.015	117	−2.478	116	0.015
ATTb	4.06	0.775	115	4.17	0.628	115	−1.834	114	0.069
SN	2.89	0.678	115	2.93	0.587	115	−0.757	114	0.451
PBC	3.51	1.208	91	3.73	1.130	91	−1.581	90	0.117
INT	1.66	0.949	91	1.9	0.987	91	−2.167	90	0.033
**Adolescents Control Group**									
**Constructs**	**Before**			**After**					
	**M**	**SD**	**N**	**M**	**SD**	**N**	**t**	**df**	**Sig.** **(2-Tailed)**
ATTd	3.85	0.902	106	3.84	0.947	106	0.061	105	0.952
ATTb	4.19	0.652	106	4.25	0.660	106	−1.276	105	0.205
SN	2.95	0.517	106	3.08	0.517	106	−2.239	105	0.027
PBC	3.77	1.164	74	3.99	0.855	74	−1.608	73	0.112
INT	1.83	1.136	74	1.79	1.064	74	0.353	73	0.725

Labels: M = mean; SD = standard deviation; ATTd = attitudes towards dance; ATTb = attitudes towards boys who dance; SN = subjective norms about boys who dance; PBC = perceived behavior control; INT = intention to join dance activities. Perceptions ranged from 1 (strongly disagree) to 5 (strongly agree).

**Table 5:** Independent samples’ test results of children and adolescents: differences before and after the SMI—experimental group.

**Before SMI**										
**Constructs**	**Children**			**Adolescents**						
	**M**	**SD**	**N**	**M**	**SD**	**N**	**t**	**df**	**Sig.** **(2-Tailed)**	**Cohen’s d**
ATTd	3.69	0.995	115	3.58	1.064	117	0.764	229	0.446	0.100
ATTb	3.97	0.691	115	4.04	0.807	117	−0.784	229	0.434	−0.103
SN	2.90	0.689	115	2.90	0.686	117	−0.043	229	0.966	−0.006
PBC	3.21	1.338	93	3.55	1.194	99	−1.846	189	0.066	−0.267
INT	1.98	1.226	93	1.78	1.076	99	1.208	189	0.229	0.174
**After SMI**										
**Constructs**	**Children**			**Adolescents**						
	**M**	**SD**	**N**	**M**	**SD**	**N**	**t**	**df**	**Sig.** **(2-Tailed)**	**Cohen’s d**
ATTd	4.05	0.892	115	3.77	1.015	117	2.214	230	0.028	0.291
ATTb	4.30	0.581	115	4.17	0.628	115	1.666	228	0.097	0.220
SN	3.13	0.640	115	2.93	0.587	115	2.394	228	0.017	0.316
PBC	3.52	1.212	83	3.67	1.162	100	−0.866	181	0.388	−0.129
INT	2.17	1.273	83	2.02	1.084	100	0.876	181	0.382	0.130

Labels: M = mean; SD = standard deviation; ATTd = attitudes towards dance; ATTb = attitudes towards boys who dance; SN = subjective norms about boys who dance; PBC = perceived behavior control; INT = intention to join dance activities. Perceptions ranged from 1 (strongly disagree) to 5 (strongly agree).

**Table 6:** Independent samples’ test results of children and adolescents: differences before and after the SMI—control group.

**Before SMI**										
**Constructs**	**Children**			**Adolescents**						
	**M**	**SD**	**N**	**M**	**SD**	**N**	**t**	**df**	**Sig.** **(2-Tailed)**	**Cohen’s d**
ATTd	3.74	0.929	98	3.85	0.902	106	−0.859	202	0.391	−0.120
ATTb	3.97	0.625	98	4.19	0.652	106	−2.460	202	0.015	−0.341
SN	3.01	0.568	98	2.95	0.517	106	0.877	202	0.381	0.123
PBC	3.57	1.271	72	3.62	1.287	85	−0.264	155	0.792	−0.042
INT	2.12	1.207	72	1.80	1.137	85	1.665	155	0.098	0.265
**After SMI**										
**Constructs**	**Children**			**Adolescents**						
	**M**	**SD**	**N**	**M**	**SD**	**N**	**t**	**df**	**Sig.** **(2-Tailed)**	**Cohen’s d**
ATTd	3.93	0.903	98	3.84	0.947	106	0.688	202	0.492	0.097
ATTb	4.27	0.537	98	4.25	0.660	106	0.245	202	0.807	0.034
SN	3.20	0.552	98	3.08	0.517	106	1.642	202	0.102	0.229
PBC	3.84	1.095	72	3.97	0.930	79	−0.750	149	0.454	−0.122
INT	2.19	1.150	72	1.81	1.095	79	2.052	149	0.042	0.331

Labels: M = mean; SD = standard deviation; ATTd = attitudes towards dance; ATTb = attitudes towards boys who dance; SN = subjective norms about boys who dance; PBC = perceived behavior control; INT = intention to join dance activities. Perceptions ranged from 1 (strongly disagree) to 5 (strongly agree).

## Data Availability

The data will be available on request.
